# PARP1pred: a web server for screening the bioactivity of inhibitors against DNA repair enzyme PARP-1

**DOI:** 10.17179/excli2022-5602

**Published:** 2023-01-05

**Authors:** Tassanee Lerksuthirat, Sermsiri Chitphuk, Wasana Stitchantrakul, Donniphat Dejsuphong, Aijaz Ahmad Malik, Chanin Nantasenamat

**Affiliations:** 1 Research Center, Faculty of Medicine Ramathibodi Hospital, Mahidol University, Bangkok 10400, Thailand; 2 Program in Translational Medicine, Chakri Naruebodindra Medical Institute, Faculty of Medicine Ramathibodi Hospital, Mahidol University, Samut Prakan 10540, Thailand; 3 Center of Excellence in Computational Molecular Biology, Faculty of Medicine, Chulalongkorn University, Bangkok 10330, Thailand; 4 Streamlit Open Source, Snowflake Inc., USA

**Keywords:** PARP-1, DNA repair, machine learning, QSAR, webserver, cheminformatics

## Abstract

Cancer is the leading cause of death worldwide, resulting in the mortality of more than 10 million people in 2020, according to Global Cancer Statistics 2020. A potential cancer therapy involves targeting the DNA repair process by inhibiting PARP-1. In this study, classification models were constructed using a non-redundant set of 2018 PARP-1 inhibitors. Briefly, compounds were described by 12 fingerprint types and built using the random forest algorithm concomitant with various sampling approaches. Results indicated that PubChem with an oversampling approach yielded the best performance, with a Matthews correlation coefficient > 0.7 while also affording interpretable molecular features. Moreover, feature importance, as determined from the Gini index, revealed that the aromatic/cyclic/heterocyclic moiety, nitrogen-containing fingerprints, and the ether/aldehyde/alcohol moiety were important for PARP-1 inhibition. Finally, our predictive model was deployed as a web application called PARP1pred and is publicly available at https://parp1pred.streamlitapp.com, allowing users to predict the biological activity of query compounds using their SMILES notation as the input. It is anticipated that the model described herein will aid in the discovery of effective PARP-1 inhibitors.

## Introduction

Precision medicine is becoming increasingly important in treating many cancers because it can reduce side effects compared with conventional therapies (Baudino, 2015[5]). Several clinical trials have shown evidence of success, especially targeting DNA repair (Brown et al., 2017[8]). For example, an ovarian phase 2 clinical trial, in which platinum-sensitive patients were given the PARP-1 inhibitor olaparib as a maintenance treatment, showed an improvement in progression-free survival (Ledermann et al., 2012[36]). In a phase 3 OlympiA clinical trial, in which olaparib was administered as an adjuvant to BRCA1/2-mutated breast cancer patients following completion of local treatment and neoadjuvant or adjuvant chemotherapy, the treatment group exhibited significantly longer survival, free of invasive or distant disease, than the placebo group (Tutt et al., 2021[74]). Moreover, the phase 2 TOPARP-A trial showed that patients who had metastatic prostate cancer, who were no longer responding to standard treatments, and who had defects in DNA-repair genes, had a high response rate toward olaparib (Mateo et al., 2015[42]).

DNA repair is a critical cellular process that ensures the integrity of the genome, allowing the parental cell to pass genetic information on to the progeny cell. Defective DNA repair causes accumulation of genetic mutations, thus leading to carcinogenesis. However, retaining some DNA repair activities is also important for cancer survival, especially when cells are under genotoxic stress (such as radio- and chemotherapy) (Helleday et al., 2008[26]). DNA double-strand break (DSB) lesions are the most toxic form of DNA damage, which, if left unrepaired, result in cell death (Shibata and Jeggo, 2014[68]). Therefore, drugs are of interest if their mode of action leads to the accumulation of DSBs (Srivastava and Raghavan, 2015[70]). 

Poly (ADP-ribose) polymerase (PARP) is an enzyme that catalyzes the ADP-ribosylation of a specific protein, resulting in the covalent binding of a single ADP-ribose unit or polymers of ADP-ribose units (Gupte et al., 2017[24]). In humans, there are 17 members of the family, although only three (PARP-1, PARP-2, and PARP-3) are involved in DNA repair (Beck et al., 2014[6]). Among the three, PARP-1 (EC 2.4.2.30) was identified in 1963 and is the most extensively investigated DNA repair enzyme (Gupte et al., 2017[24]). By inhibiting PARP-1, DSB accumulation was induced in cancer cells deficient in *BRCA1/2*, indicating that PARP-1 is a druggable target (Mateo et al., 2019[43]). Olaparib was the first well-known PARP-1 inhibitor, and it has been used as a targeted therapy to treat ovarian, breast, prostate, and pancreatic cancer patients with *BRCA1/2* mutations (de Bono et al., 2020[14]; Fong et al., 2009[20]; Golan et al., 2019[23]; Kim et al., 2015[33]). Recently, five more PARP-1 inhibitors, rucaparib (Balasubramaniam et al., 2017[3]), niraparib (Mirza et al., 2016[47]; Scott, 2017[67]), talazoparib (Hoy, 2018[27]), fluzoparib (Li et al., 2021[38]), and pamiparib (Xu et al., 2021[79]) have been approved by the Food and Drug Administration (FDA). However, access to targeted therapy has been restricted in certain countries, particularly middle- and low-income countries, because of a lack of affordability or the capability to develop domestic pharmaceutical technology, which poses a threat to health security (Fundytus et al., 2021[21]; Ocran Mattila et al., 2021[53]). As a result, accelerating drug discovery in such countries is an important factor to minimize such risk.

The computational-aided drug design (CADD) approach significantly reduces the time and cost associated with drug discovery (Nantasenamat and Prachayasittikul, 2015[51]). With the availability of public bioactivity databases such as BindingDB (Gilson et al., 2016[22]), PubChem (Kim et al., 2016[34]), GtoPdb (Armstrong et al., 2020[2]), and ChEMBL (Mendez et al., 2019[44]), we can retrieve the bioactivity data and analyze the relationship between the chemical structures of compounds and their biological activities, termed the quantitative structure-activity relationship (QSAR) (Carracedo-Reboredo et al., 2021[10]; Nantasenamat and Prachayasittikul, 2015[51]). Developing a QSAR model involves two main steps: 1) molecular structure description; and 2) multivariate analysis to correlate molecular descriptors with observed biological activities (Nantasenamat et al., 2009[50]). The first step is to define chemical structures as numerical representations of their physicochemical properties. The second step employs statistical methods to establish the relationship between the independent variables (e.g., molecular descriptors) and the dependent variables (e.g., biological activities). As a result, the QSAR model is used to predict the effects of molecular descriptor changes on biological activities, as shown by the design of inhibitors against a variety of targets, such as antiviral (Malik et al., 2020[40]; Worachartcheewan et al., 2014[78]), anti-inflammatory (Kanan et al., 2021[31]), and anticancer (Nantasenamat et al., 2014[52]; Schaduangrat et al., 2021[66]). We constructed predictive models for drug discovery using a biological dataset of PARP-1 inhibitors.

Many studies have investigated *in silico* screening of PARP-1 inhibitors, including QSAR, molecular modeling, molecular docking, molecular dynamics simulation (MD), and proteochemometric modeling (Abbasi-Radmoghaddam et al., 2021[1]; Cortes-Ciriano et al., 2015[12]; Halder et al., 2015[25]; Li et al., 2016[37]; Revathi et al., 2021[61]). Halder and colleagues (2015[25]) used comparative *in silico* studies, including 2D-QSAR, kernel-based partial least square (KPLS) analysis, pharmacophore search engine (PHASE) pharmacophore mapping, molecular docking, molecular mechanics with generalized Born and surface area solvation (MM-GBSA) analysis, and Gaussian-based 3D-QSAR analyses on docked poses to explore the structure-activity relationship of PARP-1 inhibitors (Halder et al., 2015[25]). They used 254 compounds targeting PARP-1 from Merck Research Laboratories to conduct the analysis. They found that polar interactions play an important role to leverage the activity of PARP-1. Moreover, the positive ionizable feature of ligands also plays a key role to differentiate between highly active and inactive compounds. Revathi and colleagues (2021[61]) used 71 compounds that were phthalazinone and 4-carboxamide benzimidazole derivatives to develop ligand-based pharmacophores (Revathi et al., 2021[61]). They used Pharmacophore Alignment and Scoring Engine to identify the pharmacophore sites and later developed the ADHRR.1031 pharmacophore hypothesis as a 3D-QSAR model. Furthermore, the model was validated using 1,000,000 ligands from various databases and analyzed through virtual screening. The docking analysis revealed the importance of hydrogen bonding between Gly863 and Ser904 of PARP-1 with ligands. Additionally, hydrogen bond formation with Ser864 and π-π interaction with His862, Arg878, and His909 were also observed in the docking analysis. Sahin and Durdagi (2021[64]) aimed to identify novel piperazine-based PARP-1 inhibitors (Sahin and Durdagi, 2021[64]). They used text mining to search for molecules containing piperazine as a main scaffold from the Specs-SC database. The sorted molecules were then analyzed by molecular docking, in which the ten highest docking scores were further subjected to molecular dynamics (MD) to calculate the free binding energy using the molecular mechanics/generalized born surface area method. They identified molecule-1388 as a potential candidate compound to selectively inhibit PARP-1. This compound had crucial hydrogen bonds with Gln759 and Met890 and π-π interaction with Tyr889. Abbasi-Radmoghaddam and colleagues (2021[1]) conducted a QSAR and molecular modeling study that predicted the IC_50_ values (the concentration of inhibitor at which the enzymatic activity is reduced by half) of 1H-benzo[d]immidazole-4-carboxamide derivatives (Abbasi-Radmoghaddam et al., 2021[1]). They built a QSAR model based on the genetic algorithm-multiple linear regression (GA-MLR) and least squares-support vector machine (LS-SVM) methods. Moreover, they performed molecular docking analysis to reveal the chemical interactions between the substructure in each compound and PARP-1, as well as to calculate the free energy binding. They reported nine compounds, which given the best value of IC_50_, showed an improvement in PARP-1 inhibition of 33.394 %. Li and colleagues (2016[37]) used a molecular docking approach to screen compounds from the ZINC database against PARP-1 (Li et al., 2016[37]). Grid and amber scoring were used to calculate the area under the curve from the receiver operating characteristic. The selected compounds were further analyzed through MD. Finally, they proposed ZINC67913374 as a candidate compound to inhibit PARP-1 activity. Proteochemometry was also performed by Cortés-Ciriano and colleagues (2015[12]) to develop a model to explore the relationship between PARP inhibitors and various PARP isoforms, including PARP-1 (Cortes-Ciriano et al., 2015[12]). They used both chemical (Morgan fingerprints) and protein (binding site amino acid (AADescs) and full protein sequence (SeqDescs) descriptors as independent variables, while thermal shift values retrieved from Differential Scanning Fluorimetry (DSF) were treated as dependent variables. The models were built based on random forests, which were then further examined for the confidence intervals to understand the reliability of the predictive performance for either new compounds or PARP isoforms. Altogether, these studies show that computational approaches are useful to identify novel inhibitors of PARP-1.

In this study, we used Python-based programming to retrieve the biological activities of human PARP-1 from ChEMBL (Mendez et al., 2019[44]). We extracted a total of 2018 non-redundant compounds with known IC_50_ values. All the inhibitors were converted to 12 different molecular descriptors and further built with 12 different machine learning models. Of the 144 models, the PubChem random forest model was chosen, because it was interpretable and it robustly classified substances as active or inactive, as indicated by MCC values > 0.7 of the training and CV sets in all three sampling approaches. Additionally, the important chemical fingerprints that contributed to the constructed model were examined. In-depth analysis of the top 20 descriptors demonstrated that aromatic/heterocyclic and nitrogen-containing characteristics are important for PARP-1 inhibition. Lastly, a web server was built to make this prediction accessible in the public domain. This will accelerate the discovery of new and diverse inhibitors against PARP-1.

## Materials and Methods

### Data compilation and curation

The dataset of PARP-1 (ChEMBL ID: CHEMBL3105) inhibitors was compiled using data from the ChEMBL database, release 29 (Mendez et al., 2019[44]), which includes an initial set of 5094 bioactivity data points and 3738 compounds. The data were retrieved through a Python-based library (https://pypi.org/project/chembl-webresource-client/) which enables users to cache all results in the local file system for faster retrieval (Davies et al., 2015[13]). The IC_50 _values, containing 2815 data points and 2429 compounds, were chosen for further curation. Because the purpose of this study was to create a classification model for PARP-1 inhibition, we defined active as ≤ 1 µM (n = 1720) and inactive as ≥ 10 µM (n = 298). The intermediates with concentrations ranging between 1 and 10 µM were discarded (n = 334). Finally, we obtained 2018 non-redundant and curated active and inactive compounds for further analysis.

### Molecular descriptor analysis

The PaDEL-Descriptor software was used to calculate molecular fingerprints for each compound in the dataset (Yap, 2011[82]). As previously described by Malik and colleagues (2020[40]), molecular fingerprints are numerical values that represent both qualitative and quantitative chemical structures (Malik et al., 2020[40]). Thus, they are crucial for QSAR studies. The software computes 12 types of fingerprints which belong to nine classes, namely, Atom Pairs 2D, CDK, CDK extended, CDK graph only, E-state, Klekota-Roth, MACCS, PubChem, and Substructure. Moreover, Atom Pairs 2D, Klekota-Roth, and Substructure are available in two versions. The first version indicates the presence or absence of the descriptors using the values 1 and 0, while the second version indicates the descriptor's frequency value. The structures in SMILES format were pre-processed by removing salt, detecting aromaticity, standardizing nitro groups, and standardizing tautomers, before being subjected to molecular fingerprint calculation.

### Data filtering

During the feature selection process, low variance variables were not useful for the model's predictive capability. Therefore, constant and near constant variables were omitted from the selection of fingerprint descriptor sets to reduce model complexity and bias. The constants of the fingerprint descriptors were calculated using a standard deviation (SD) of 0.1 as a cut-off value. Thus, variables with SD values of less than 0.1 were selected for further analysis.

### Data splitting for model construction

The Kennard-Stone algorithm was used to divide the data into an 80/20 ratio (Kennard and Stone, 1969[32]), of which 80 % was assigned as an internal set (1614 compounds, active = 1380, inactive = 234) and the remaining 20 % was used as an external set (404 compounds, active = 340, inactive = 64) to validate the model. The internal dataset was further divided into balanced and imbalanced datasets and used as the training dataset, which was subjected to five-fold cross-validation.

### Statistical analysis

We present chemical descriptors of each molecule according to the previous study by Schaduangrat and colleagues (2021[66]). Briefly, this uses six common descriptive statistical parameters: minimum (Min), first quartile (Q1), median, mean, third quartile (Q3), and maximum (Max). All the parameters were visualized as a box plot using the seaborn and matplotlib data visualization packages in Python. Lipinski's rule-of-five parameters were compared between active and inactive groups using the Mann-Whitney *U* test, with *p* < 0.05 indicating a significant difference.

### Multivariate analysis

Twelve machine learning classification models were constructed from the internal dataset: decision trees, extra trees, Gaussian Naive Bayes, Gaussian process, gradient boosting, K-neighbors, light gradient boosted machine, multi-layer perceptron, quadratic discriminant analysis, random forest, C-support vector, and extreme gradient boosting. The model construction was developed using the scikit-learn library (Pedregosa et al., 2011[59]) in Python. Each type of model had different characteristics to determine the relationship between the dependent variables and the independent variables. Gradient boosting, random forest, extra trees, light gradient boosted machine, and extreme gradient boosting were grouped as ensemble methods, which generate many models and combine them to get the best model. Multi-layer perceptron was part of the neural network, which was considered a black box model and could not be interpreted. Decision tree was used to learn simple decision rules retrieved from the data features. K-neighbors is a type of instance-based learning in which the classification of certain data is based on most of its nearest neighbors. Support vector machine draws a hyperplane to separate two or more classes in the best possible manner. The Gaussian process uses a Gaussian distribution to fit random points of data, whereas quadratic discriminant analysis estimates the means and covariances from the data and assigns a new observed data point to the class with the greatest likelihood. Lastly, Gaussian Naive Bayes assumes each feature follows Gaussian distribution, calculates the probability from each feature at a given class, and multiplies all the probabilities of each feature.

### Model validation

We used a variety of statistical parameters to evaluate the performance of the models, including true positives (TP), true negatives (TN), false positives (FP), and false negatives (FN). The model's fitness was determined using the following statistical parameters: overall prediction accuracy (Ac), sensitivity (Sn), specificity (Sp), and Matthews correlation coefficient (MCC).


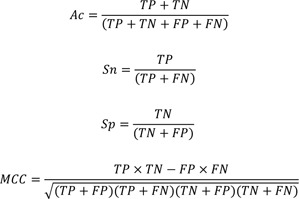


### Applicability domain analysis

To estimate the chemical space in which the model can make reliable and accurate predictions for compounds based on similarity with the compounds on which the model was constructed, we used the PCA bounding box to determine the applicability domain (AD) of compounds from the training (internal) and test (external) sets. Compounds that fall inside the AD of the model are typically predicted reliably.

### Reproducibility research

The data and code used in the study are deposited on GitHub at https://github.com/tlerksuthirat/data_driven_PARP1.

### Development of the PARP-1 web server

The best predictive model was exported as model.pkl and is used in the deployed web server developed in Python using Streamlit version 1.12.0. Particularly, the Streamlit web app accepts the input SMILES notation of query molecule and converts this into an image file of the 2D chemical structure via rdkit-pypi version 2022.3.5. Subsequently, the SMILES notation is used to compute the PubChem molecular fingerprint using padelpy version 0.1.10. The best machine learning model, which was built using the random forest algorithm with scikit-learn version 1.0.2, is applied on the computed fingerprint of the query molecule where the bioactivity is predicted. The PARP1pred web app is publicly available at https://parp1pred.streamlit.app/ while the data and code used for building this app is deposited on GitHub at https://github.com/dataprofessor/parp1.

## Results and Discussion

**Figure 1:**
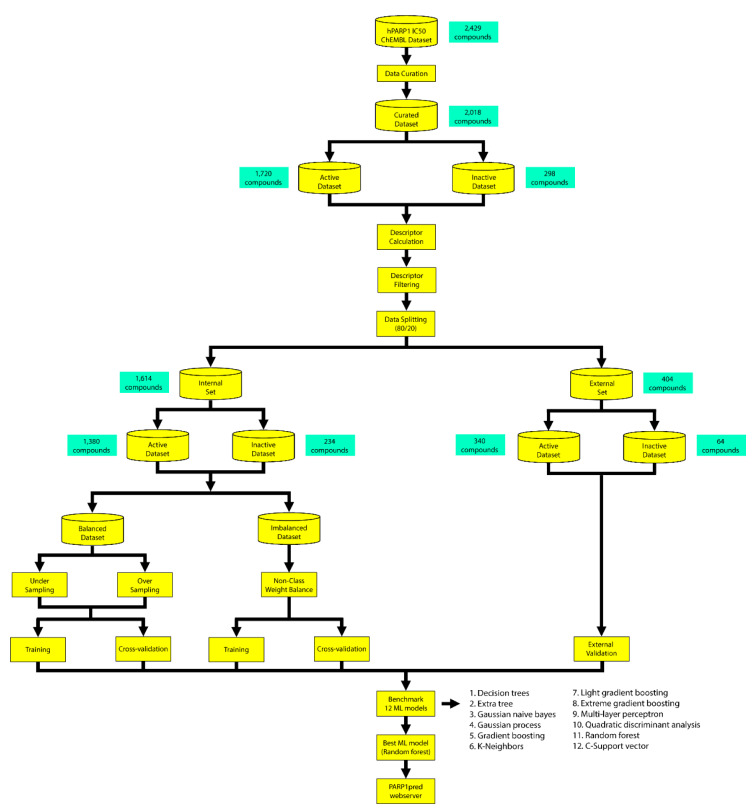
Overall workflow of the development of the webserver for PARP-1 inhibitors

The entire workflow for constructing the model is summarized in Figure 1(Fig. 1).

### Chemical space analysis

**Figure 2:**
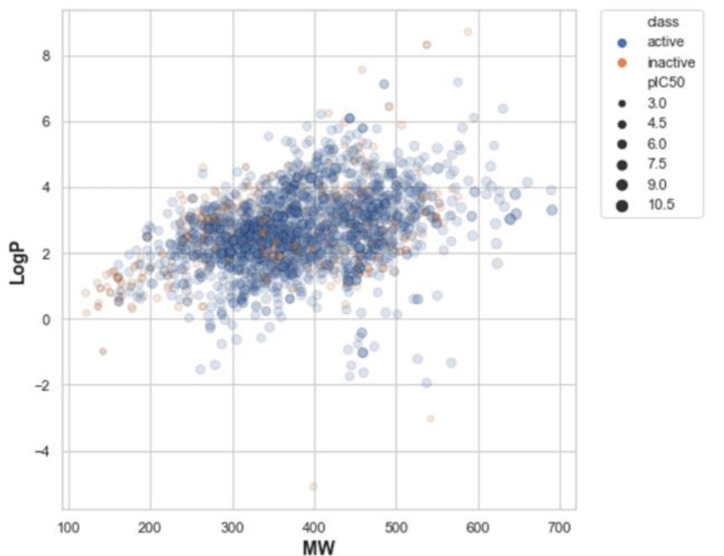
Illustration of the relationship between molecular weight (MW) and Ghose-Crippen-Viswanadhan octanol-water partition coefficient (LogP). Blue and orange represent active and inactive compounds. The size of the circle refers to the pIC_50_ value, which is the negative logarithmic of the IC_50_ concentration (nM).

**Figure 3:**
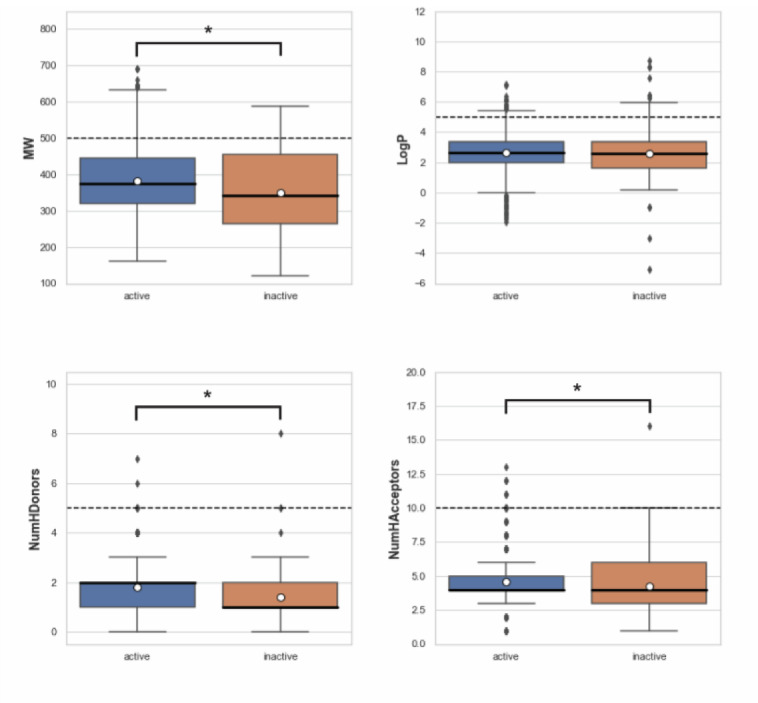
Box plots of Lipinski's rule-of five descriptors comparing between active and inactive groups. The dashed line represents cut-off values indicating drug-like molecules: molecular weight (MW) < 500, Ghose-Crippen-Viswanadhan octanol-water partition coefficient (LogP) < 5, number of hydrogen bond donors (NumHDonors) < 5, number of hydrogen bond acceptors (NumHAcceptors) < 10. A circle represents the mean, and an asterisk indicates a significant difference between two groups (*p* < 0.05).

The aim of performing chemical space analysis between active and inactive compounds is to understand the difference in chemical characteristics between two groups. We first explored the relationship between molecular weight (MW) and the Ghose-Crippen-Viswanadhan octanol-water partition coefficient (LogP), as shown in Figure 2(Fig. 2) (Wildman and Crippen, 1999[77]). LogP is a lipophilic descriptor that can be used to determine the permeability of molecules to the cell membrane, thereby indicating their drug-likeness molecule (van de Waterbeemd, 2008[76]). Next, Lipinski's rule-of-five (Ro5) descriptors were employed to investigate the difference in chemical features between active and inactive compounds, as shown in Figure 3(Fig. 3). The Ro5 are composed of four parameters, namely MW (< 500 kDa), LogP (< 5), the number of H-bond donors (NumHDonors < 5), and the number of H-bond acceptors (NumHAcceptors < 10) (Lipinski et al., 2001[39]). If any compounds have values out of range for two parameters, they are likely to have poor absorption or permeability, and thus a higher rate of drug development failure. As illustrated in Figure 2(Fig. 2), most compounds clustered between 300 and 500 MW with a LogP of 2-4. Moreover, the Ro5 analysis and statistical analysis revealed that most of the active and inactive compounds following the Ro5 as illustrated by the box plots were under the cut-off values (dashed line, Figure 3(Fig. 3)). The Mann-Whitney *U *test found a significant difference in MW, NumHDonors, and NumHAcceptors between active and inactive molecules, but no difference in LogP. Active molecules had a higher MW, NumHDonors, and NumHAcceptors than inactive molecules, as demonstrated by the circle in the boxplot (Figure 3(Fig. 3)). The mean ± SD of MW in the active and inactive groups were 381.66 ± 87.93 and 349.35 ± 119.32, respectively. NumHDonors had a mean ± SD of 1.80 ± 0.82 for active molecules and 1.39 ± 0.79 for inactive molecules, whereas the mean SD for NumHAcceptors was 4.56 ± 1.53 for active molecules and 4.22 ± 2.06 for inactive molecules. Between the active and inactive molecules, the logP value was 2.66 ± 1.17 for active molecules and 2.61 ± 1.52 for inactive molecules.

### QSAR modeling

To develop a robust QSAR model, we followed the guidelines of the Organization for Economic Co-operation and Development (OECD, 2014[54]). Briefly, a robust model should include, at least: 1) a defined endpoint for the dataset; 2) an unambiguous learning algorithm; 3) a defined applicability domain of the QSAR model; 4) appropriate measures of goodness-of-fit, robustness, and predictability; and 5) mechanistic interpretation of the QSAR model. Thus, to develop interpretable QSAR models, the molecular fingerprints indicated in Table 1(Tab. 1) were calculated using the PaDEL-Descriptor software, from which three fingerprints (PubChem, Substructure, and Klekota-Roth) are readily interpretable.

We constructed 12 machine learning models from 12 molecular fingerprints to determine which model gave the best performance and was the most robust and interpretable. Because our imbalanced data contained more active compounds (n = 1720) than inactive compounds (n = 298), we compared the models generated from both balanced and imbalanced approaches. Prior to data splitting, we reduced the dimensionality of the data by selecting the fingerprint that rendered SD < 0.1. The data were split into external and internal sets in an 80:20 ratio. The internal dataset (n = 1614), which contained 1380 active and 234 inactive compounds, was further divided into balanced and imbalanced datasets. For the balanced dataset, the models were created based on two methods: 1) undersampling, which randomly selected the majority class equal to the number of the minority classes; 2) oversampling, which amplified the number of minority classes equal to the number of the majority class. 

**Figure 4:**
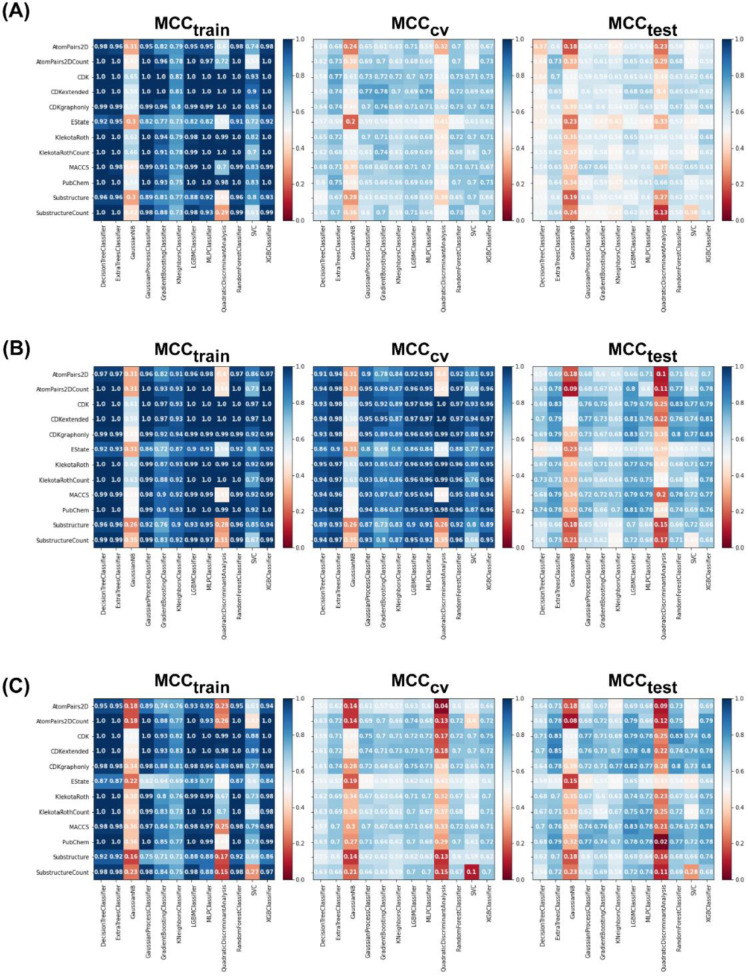
Heat maps of the MCC values of the training, CV, and test sets for each data sampling approach. (A) Balanced undersampling, (B) balanced oversampling, and (C) imbalanced non-class weight. Abbreviations: MCC, Matthews correlation coefficient; CV, cross-validation; gaussianNB, Gaussian Naive Bayes; LBMC, light gradient boosted machine; MLP, multi-layer perceptron; SVC, C-support vector; XGB, extreme gradient boosting

**Figure 5:**
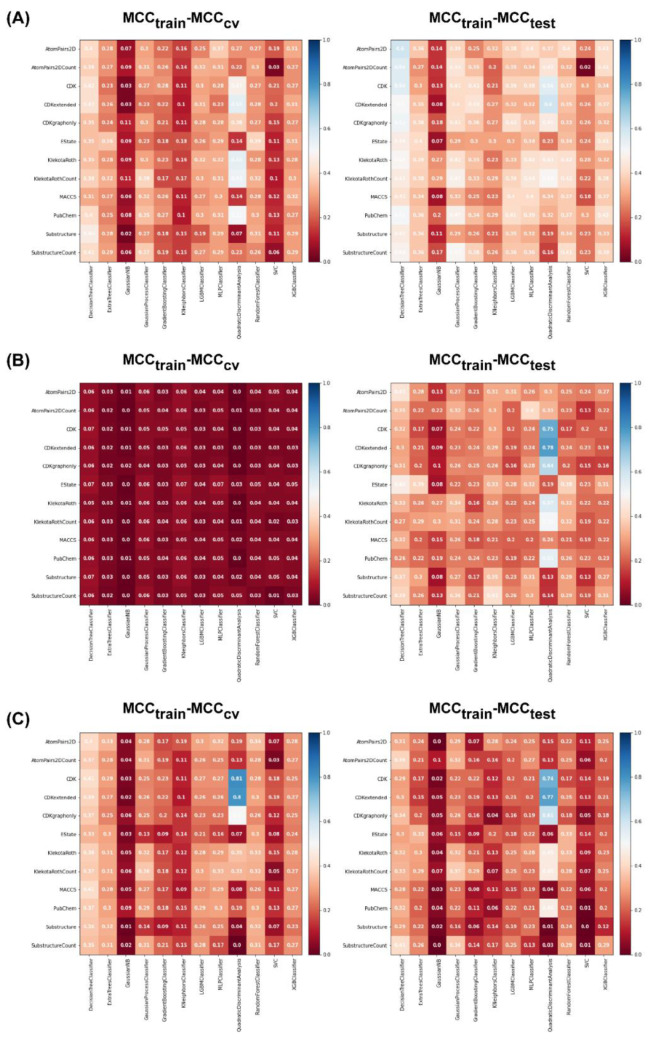
Heat maps of MCC_train_−MCC_CV_ and MCC_train_−MCC_test_ for each data sampling approach. (A) Balanced undersampling, (B) balanced oversampling, (C) imbalanced non-class weight. Abbreviations: MCC, Matthews correlation coefficient; CV, cross-validation; gaussianNB, Gaussian Naive Bayes; LBMC, light gradient boosted machine; MLP, multi-layer perceptron; SVC, C-support vector; XGB, extreme gradient boosting

For the non-class weight balance of an imbalanced dataset, the data were randomly selected to develop the model without consideration of the ratio between major and minority classes. Figures 4(Fig. 4) and 5(Fig. 5) demonstrate the heat maps of MCC_train_, MCC_CV_, MCC_test_, MCC_train−CV_, and MCC_train−test_ for each fingerprint, machine learning model, and sampling approach. 

**Figure 6:**
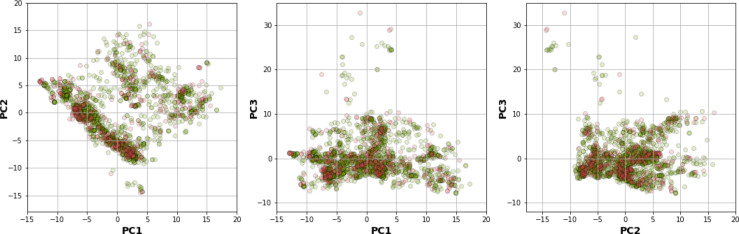
Plot of PCA scores for applicability domain analysis. The score plot indicates the distribution of chemical space of the internal (green) and external (red) datasets, which were used to determine the applicability domain of the PARP-1 inhibitors dataset.

Results showed that a balanced oversampling approach yielded the best value-most of the MCC_train_ and MCC_CV_ values were more than 0.8. Moreover, most of the MCC_test_ values of oversampling were more than 0.7. The values of MCC_train−CV_ in the oversampling group were lower than 0.2, whereas the values of MCC_train−test_ in both balanced oversampling and imbalanced non-class weight were generally better than balanced undersampling, as the MCC_train−test_ values of undersampling were mostly greater than 0.3. As a result, we considered the oversampling approach as a good candidate to compare the performance among each model and fingerprint. Figure 4B(Fig. 4) demonstrates that Gaussian Naive Bayes and quadratic discriminant analysis did not yield acceptable MCC values (< 0.7) for all fingerprints. We further selected random forest (RF) over other machine learning methods because relevant features were able to be observed and the model was easily interpretable. As mentioned in the Methods section, RF is an ensemble method that has a root node as a starting point and splits into an N number of decision trees to learn the inherent patterns from the input data (Breiman, 2001[7]). Following a thorough examination of all MCC values for the interpretable fingerprints- PubChem, Substructure, and Klekota-Roth- the result suggested that a model based on PubChem was a good candidate. This was demonstrated by the MCC values for PubChem in the training, cross-validation, and test sets of 1, 0.96, and 0.74, respectively, whereas the MCC values for Substructure and Klekota-Roth in the test set were 0.66 and 0.68, respectively. As a result, the RF model that was developed using the oversampling approach from the PubChem fingerprint was the best option for model interpretation. Furthermore, as indicated in Figure 6(Fig. 6), the applicability domain was determined using the PubChem fingerprint as the input for PCA analysis. A total of 2018 compounds were split into two subsets, which consisted of internal (80 %) and external (20 %) datasets using the Kennard-Stone algorithm (Kennard and Stone, 1969[32]). The internal set was used as the training dataset, subjected to random sampling, and the predictive model was constructed with five-fold cross-validation. The result showed that the chemical space distribution of the external dataset fits well with the internal dataset, indicating that the applicability domain was well defined for the QSAR-based classification model. 

### Mechanistic interpretation of feature importance

**Figure 7:**
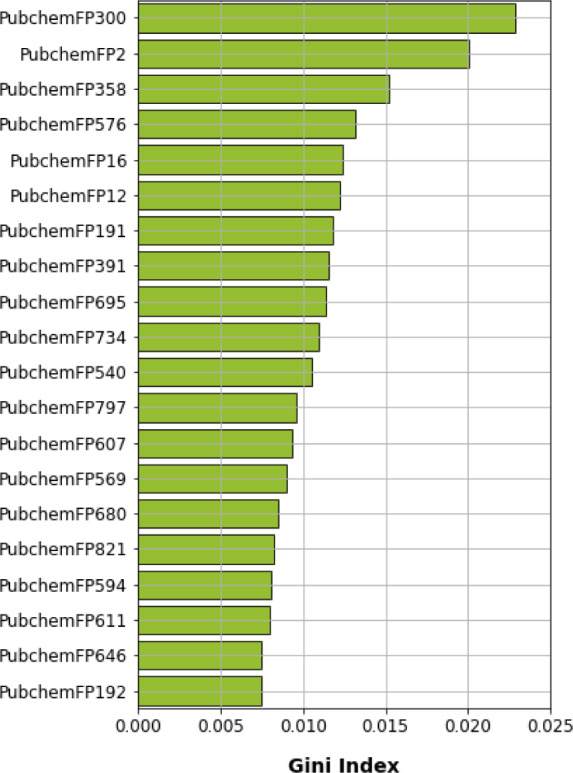
Feature importance plot as rationalized by Gini index obtained from random forest model using oversampling

To gain a better understanding of the mechanisms underlying PARP-1 activity and the significance of the features used to develop a PARP-1 activity predictability model using RF, the mean decrease of the Gini index was used to rank the importance of the PubChem feature descriptors. Measuring feature importance in RF can be evaluated by the mean decrease accuracy and the mean decrease in Gini; however, the latter gives more robust results (Calle and Urrea, 2010[9]). Thus, we selected the top 20 PubChem substructures with the highest Gini index, illustrated in Figure 7(Fig. 7), and their corresponding substructure descriptions are shown in Table 2(Tab. 2). We grouped the functional groups of the PubChem fingerprints into four classes: 1) aromatic, cyclic/heterocyclic, and ring counts; 2) nitrogen-containing, consisting of hydrazine, amine, imine, and amide; 3) atom counts; and 4) ether, aldehyde, and alcohol. However, some PubChem fingerprints had more than one feature; for example, PubChemFP695 had aldehyde and amine functional groups, and PubChemFP821 had cyclic and amine functional groups.

### Aromatic, cyclic/heterocyclic, and ring count functional groups

The fingerprints belonging to these groups consisted of PubChem191, PubChem734, PubChem797, PubChem821, and PubChem192. PubChem192 was on the lowest rank of the top 20 and it was not specified whether it was aromatic- or heteroatom-containing, but it must have a ring size of six for at least three rings. Thus, the aromatic (PubChemFP734) and cyclic (PubChem191, PubChem797, and PubChem821) moieties overlapped with PubChem192. Based on aromatic, cyclic/heterocyclic, and ring counts, PubChem191, PubChem797, and PubChem821 were at the 7^th^, 12^th^, and 16^th^ positions of the top 20. Taking a closer look at our post-processing dataset (2018 compounds), there were 35 compounds in total containing all three fingerprints, of which 34 compounds were considered active. Moreover, a total of 33 compounds contained both cyclic (PubChem191, PubChem797, and PubChem821) and aromatic moieties (PubChemFP734), and all of them were active. This meant that aromatic and cyclic/heterocyclic functional groups with a ring size equal to six or more than two were the important features of the active compounds. The first generation of PARP-1 inhibitors was designed to mimic the benzamide scaffold of NAD^+^ (Steffen et al., 2013[71]). Later the efficacy was improved by using quinazolinone as a scaffold to synthesize PARP-1 inhibitors (Malyuchenko et al., 2015[41]). Inhibitors derived from those two scaffolds contain both the aromatic and cyclic/heterocyclic moieties and play an important role in the NAD^+^ binding pocket. The aromatic ring forms π-π interactions with the tyrosine residues in the NAD^+^ binding pocket, and both the aromatic ring and cyclic/heterocyclic moieties form hydrophobic interactions with the hydrophobic residues in the NAD^+^ binding pocket. The crystal structure of human PARP-1 revealed a hydrophobic interaction between the quinazolinone part of the FR257517 inhibitor and the phenyl ring of Tyr907 and a CH-π interaction with Cβ of Tyr869 (Kinoshita et al., 2004[35]). Moreover, docking analysis between PARP-1 and tricyclic compounds containing a non-aromatic A-ring demonstrated the fit within the NAD^+^ binding pocket, even though the non-aromatic A-ring was not flat (Park et al., 2010[56]). Most of the active compounds reported herein had IC_50_ values ranging from 0.013-0.695 µM. It should be noted that PubChem191 was in the highest rank among the aromatic, cyclic/heterocyclic, and ring counts groups. This could be explained by the nitrogen in the non-aromatic moiety of the inhibitors contributing to hydrogen bonds forming with the glycine in the NAD^+^ binding pocket. The crystal structure of PARP-1 conjugated with FR257517 revealed three hydrogen bonds, one from the NH of the quinazolinone part of FR257517 to Gly863-C=O (Kinoshita et al., 2004[35]). In addition, cyclic benzamide derivatives increased potency in PARP-1 and led to the optimization of novel PARP-1 inhibitors. Steinhagen and colleagues (2002[72]) reported that core variations within the cyclohexene moiety of PubChem191 affected the potency of inhibitors (Steinhagen et al., 2002[72]). Moreover, the study demonstrated that substitution of the 3,6-dihydro-2-thiopyrane subunit yielded a three- to tenfold increase in potency compared with the cyclohexenyl moiety.

### Nitrogen-containing functional groups, including hydrazine, amine, imine, and amide

This class of functional groups possessed the largest number of fingerprints, including hydrazine (PubChemFP300), amine (PubChemFP358, PubChemFP391, PubChemFP695, PubChemFP540, PubChemFP607, PubChemFP569, PubChemFP821, and PubChemFP611), amide (PubChemFP646), and imine (PubChemFP576). There were two fingerprints in this group, PubChemFP695 and PubChemFP821, also containing aldehyde and cyclic functional groups, respectively.

PubChemFP300 was in the first rank of important fingerprints based on all features. This is because PubChemFP300 is part of the basic scaffold during PARP-1 inhibitor development (Ferraris, 2010[19]). Banasik and colleagues (1992[4]) introduced pthalazine derivatives and analogues as part of the development of PARP-1 inhibitors (Banasik et al., 1992[4]). Moreover, Xu and colleagues (2014[80]) synthesized a series of compounds which contained tetraaza phenalen-3-one as a main scaffold to inhibit PARP-1 (Xu et al., 2014[80]). The compounds sensitized tumor cells to ionizing radiation and temozolomide. Ji and colleagues (2015[29]) used phthalic hydrazide as a pharmaceutical scaffold to synthesize novel PARP-1 inhibitors (Ji et al., 2015[29]). Another study produced novel PARP-1 inhibitors by fusing a pyrazolo pyridin-2-one to a non-aromatic heterocycle or carbocycle. These resulted in a vast variety of IC_50_ values, ranging from 0.002 to >10 µM (Moree et al., 2008[48]).

As well as PubChemFP300, another four fingerprints were within the top ten important features: PubChemFP358 (3^rd^ rank), PubChemFP576 (4^th^ rank), PubChemFP391 (8^th^ rank), and PubChemFP695 (9^th^ rank). PubChemFP358 is part of the benzamide scaffold, thus making it critical for PARP-1 inhibitor synthesis because this scaffold mimics the NAD^+^ substrate. This scaffold has been maintained through all generations of PARP-1 synthesis (Malyuchenko et al., 2015[41]). As previously mentioned, the crystal structures revealed that NH in the quinazolinone scaffold of FR257517 forms a hydrogen bond with the Gly863-C=O that is required for the inhibitor to remain in the NAD^+^ binding pocket (Kinoshita et al., 2004[35]). Moreover, PubChemFP358 is part of the pendant fluorobenzyl group that participates in the adenine-ribose binding pocket within the NAD^+^ binding site (Pescatore et al., 2010[60]).

PubChemFP576 is part of the pyridine and pyrimidine moieties. Moree and colleagues (2008[48]) fused a pyrazolo pyridin-2-one to a non-aromatic heterocycle or carbocycle to generate novel PARP-1 inhibitors (Moree et al., 2008[48]). The fused structures were designed based on the observation that pyrazolo pyridin-2-one showed a similar binding mode between chicken PARP-1 (PDB: 1PAX) and the Parke-Davis/Pfizer inhibitor. Ferraris and colleagues (2003[18]) synthesized a series of aza-5[*H*]-phenanthridine-6-inhibitors where nitrogen atoms were introduced to the 5[*H*]-phenanthridin-6-one core at different positions to compare the potency (Ferraris et al., 2003[18]). Moreover, this fingerprint was part of the tetraaza phenalen-3-one (Xu et al., 2014[80]), 4-benzyl-2*H*-phthalazin-1-one (Menear et al., 2008[45]), and 4-[4'-fluoro-3'-(piperazine-1'-carbonyl)benzyl]-2H-phthalazin-1-one cores (Zmuda et al., 2015[87]). Torrisi and colleagues (2010[73]) demonstrated that introduction of 3-pyridyl to a hexahydrobenzonaphthyridinone pharmacophore resulted in metabolic stability (Torrisi et al., 2010[73]).

PubChemFP391 represents the tertiary amines that Ferraris and colleagues (2003[17]) added to the partially saturated aza-5[*H*]-phenanthridine-6-ones to increase aqueous solubility (Ferraris et al., 2003[17]). Moreover, it is part of the optimal nitrogen substituent of the hexahydrobenzophthyridinone pharmacophore to synthesize diverse ranges of PARP-1 inhibitors that was synthesized by Torrisi and colleagues (2010[73]). Pescatore and colleagues (2010[60]) synthesized a series of pyrrolo[1,2-a]pyrazin-1(2*H*)-one to inhibit PARP-1 (Pescatore et al., 2010[60]). Additionally, the same study revealed that the pyrrolo[1,2-a]pyrazin-1(2*H*)-one scaffold exhibited good potency and inhibited *BRCA*-deficient tumor cells. Rhee and colleagues (2009[62]) used isoquinolinone-based tetracycles as the main scaffold to develop PARP-1 inhibitors (Rhee et al., 2009[62]). Based on this fingerprint, some of the compounds from this study exhibited an IC_50_ lower than 1 µM. Zhou and colleagues (2017[85]) made a group of compounds called fused tetra- or penta-cyclic compounds, in which one part of the ring had a tertiary amine as a spacer to link other substituents, that showed diverse ranges of enzymatic activity (Zhou et al., 2017[85]).

PubChemFP695 overlapped with both PubChemFP358 and PubChemFP191, which are important for the NAD^+^ binding pocket. Moreover, PubChemFP695 was part of tricyclic derivative PARP-1 inhibitor synthesis (Myung-Hwa et al., 2014[49]), and substituents participated in the adenine-ribose (AD) binding site within the NAD^+^ binding pocket (Scarpelli et al., 2010[65]). PubChemFP695 is a component of proline derivatives and contributes to lipophilicity, which is necessary for cell permeability (Dunn et al., 2012[15]). This was confirmed by introducing the polar carboxylic acid moiety to proline derivatives, resulting in less cell-based activity. Moreover, PubChemFP695 also overlapped with PubChemFP391, making this fingerprint part of the AD binding site.

Collectively, this suggests that nitrogen-containing fingerprints are important in model construction.

### Ether, aldehyde, and alcohol functional groups

One fingerprint, PubChem695, which contained both aldehyde and amine functional groups, is categorized in this class and has been discussed previously. The remaining fingerprints falling into this class, PubChem680 (15^th^ rank) and PubChem594 (17^th^ rank), were not ranked in the top ten important features. Based on our curated dataset (n = 2018), few compounds contained these fingerprints: PubChem680, n = 714 (18^th^ rank); and PubChem594, n = 468 (18^th^ rank). PubChem680 is composed of alkane and alcohol functional groups and participates in the nicotinamide-ribose (NI) and AD binding sites within the NAD^+^ binding pocket. The study led by Ferraris and colleagues (2003[18]) replaced the C=O of the amide group from the benzamide scaffold with C-OH, which resulted in IC_50_ values ranging from 14-0.042 µM (Ferraris et al., 2003[18]). This suggests that OH could be able to maintain a hydrogen bond within the NAD^+^ binding pocket. Additionally, this fingerprint served as an o-linked spacer between two distinct pharmacophores, one of which was responsible for the NI binding site and the other for the AD binding site, as demonstrated by Park and colleagues (2010[56]) via the synthesis of a series of 1,2-dihydro-4H-thiopyrano[3,4-c]quinolin-5(6H)-one derivatives (Park et al., 2010[56]). As part of the AD binding site, this fingerprint also overlapped with PubChemFP695, which contributes to aqueous solubility and cellular permeability, as previously mentioned.

PubChemFP594 is part of the pyran and was found to play roles in both the NI and AD binding sites within the NAD^+^ binding pocket. Several studies have used pyran as part of the scaffold. For example, introducing a dihydropyran to the A-ring caused the derivatives to be more polar but less potent toward PARP-1 inhibition (Shultz et al., 2013[69]). Xu and colleagues (2014[81]) filed the patent on the synthesis of diazabenzo[de]anthracen-3-one derivatives that contain pyran as part of the tri-cyclic ring (Xu et al., 2014[81]). All the compounds reported in this study were categorized as active compounds. Conversely, the patent filed by Cheung and colleagues (2015[11]) revealed mostly inactive compounds against PARP-1 (Cheung et al., 2015[11]). For the AD binding site, this fingerprint participated in phenyl derivative substituents, as demonstrated by Orvieto and colleagues (2009[55]) when they introduced methyl groups to the aromatic ether (Orvieto et al., 2009[55]). They found that this improved the inhibitory effect compared with its parental phenyl. As previously mentioned, PubChemFP594 also functionally overlapped with PubChem680, as part of the o-linked spacer between two binding modes of pharmacophores.

### Structural interpretation

**Figure 8:**
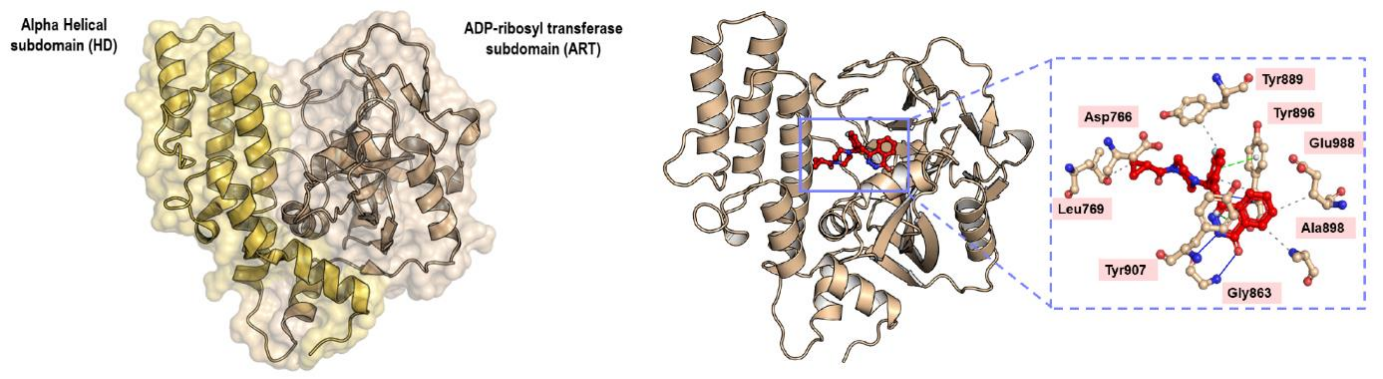
Crystal structure of the catalytic domain of PARP-1 (PDB ID 1UK0) and the interaction network between PARP-1 and olaparib (PDB ID 7KK4). The alpha-helical subdomain (HD) is shown in light orange color while the ADP-ribosyl transferase subdomain (ART) is shown in wheat color. Hydrogen forming network (blue solid line), π-π (green dashed line), and hydrophobic (grey dashed line) interactions between key amino acids within the nicotinamide binding site and olaparib

PARP-1 has three important domains: 1) the DNA binding domain, 2) the catalytic domain, and 3) the nuclear acceptor protein (Ferraris, 2010[19]). The catalytic domain is subdivided into: 1) the helical domain (HD), and 2) the ADP-ribosyl transferase (ART) domain, as illustrated in Figure 8(Fig. 8) (Patel et al., 2012[58]). Most of the compounds were synthesized to inhibit the catalytic domain that consists of three subsites: 1) the nicotinamide-ribose binding site (NI), 2) the phosphate binding site (PH), and 3) the adenine-ribose binding site (AD), and the inhibitors were designed to mimic the nicotinamide scaffold of NAD^+^ (Kinoshita et al., 2004[35]). Thus, all generations of PARP-1 inhibitors have maintained the basal chemical interaction network between the inhibitors and the key amino acids within the NI binding site (Malyuchenko et al., 2015[41]). These key amino acids include Gly863 (nitrogen of the α-amine) and Ser904 (oxygen of the R-group) forming hydrogen bonds with either C=O or C-OH of inhibitors. The oxygen of the carboxyl group of Gly863 forms a hydrogen bond with either the nitrogen-containing ring of inhibitors or the NH group of the nicotinamide scaffold, whereas the hydrogen of the amino group of Gly863 forms a hydrogen bond with either the C=O or C-OH of the inhibitors, as illustrated in Figure 8(Fig. 8). Additionally, π-π and hydrophobic interactions between the side chain of Tyr896 and Tyr907 in PARP-1 and either the cyclic or aromatic ring of inhibitors contribute to the NI binding site, as shown in Figure 8(Fig. 8). These interactions were shown by the co-crystallization of chicken PARP-1, which is highly conserved with human PARP-1 (sequence identity and similarity, 79 % and 89 %, respectively), with three different inhibitors: 6-amino-benzo[de]isoquinoline-1,3-dione (4ANI), 3-methoxybenzamide (3MBA), and 8-hydroxy-2-methyl-3-hydro-quinazolin-4-one (NU1025) (Kinoshita et al., 2004[35]; Ruf et al., 1998[63]). The importance of the chemical interaction network has been confirmed through site-directed mutagenesis on human PARP-1. Ruf and colleagues (1998[63]) demonstrated that G863A, Y896N, and Y907N reduced PARP-1 activity to 70 %, 15 % and 1.1 %, respectively, compared with wildtype (Ruf et al., 1998[63]).

To improve the potency of PARP-1 inhibitors, because the NI binding site is found in other NAD^+^ binding proteins, the development of PARP-1 inhibitors was extended to use the AD binding site to increase the selectivity of PARP-1 inhibition. In particular, this helps to differentiate between PARP-1 and PARP-2, which share very high similarity at the active site, and double knockout of PARP-1 and PARP-2 is lethal during embryogenesis (Ménissier de Murcia et al., 2003[46]). PARP-2 knockout in mice also demonstrated a role in maintaining the genetic integrity of hematopoietic stem/progenitor cells (Farrés et al., 2013[16]). Cross-reactivity of inhibitors with PARP-2 could therefore have significant side-effects.

The amino acids making up the AD binding site include Glu763, Asp766, Asn767, Leu769, Asp770, His862, Ser864, Asn868, Ile872, Gly876, Ile877, Arg878, and Ala880, as defined by several co-crystal structures (Kinoshita et al., 2004[35]; Patel et al., 2012[58], 2014[57]). Glu763, Asp766, Asn767, and Asp770 are part of the helical domain which uncoils upon DNA-binding activation, thus enabling inhibitors to insert into the catalytic pocket (van Beek et al., 2021[75]). Ishida and colleagues (2006[28]) used structure-based drug design to understand the different interactions of inhibitors between PARP-1 and PARP-2 (Ishida et al., 2006[28]). They discovered that two chemical frameworks, quinazolinone and quinoxaline derivatives, fit the AD binding site differently and inhibit PARP-1 and PARP-2, respectively. Zhao and colleagues (2017[84]) modified the spacer and the *N*-Boc-pyrrolidin-3-yl subunit of a quinazoline-2,4(1*H*,3*H*)-dione derivative to adjust the interaction within both the spacer and the AD binding site (Zhao et al., 2017[84]). Moreover, Zhou and colleagues (2021[86]) exploited the unique AD binding site between PARP-1 and PARP-2 to generate a series of quinazoline-2,4(1*H*,3*H*)-dione derivatives with a variety of substituted cyclic amines (Zhou et al., 2021[86]). They reported that compound 24, which had an (*R*)-3-ethyl piperazine ring, showed high enzymatic potency and selectivity toward PARP-1. This compound also demonstrated an acceptable pharmacokinetic profile and reduced tumor growth in xenograft and orthotopic models of breast cancer and glioblastoma, respectively. Co-crystallization of PARP-1 with compounds 4 (PDB ligand ID 6WZ) and 6 (PDB ligand ID 6X2) demonstrated a favorable hydrophobic interaction of either the methyl or ethyl substituent on the piperazine ring with the key amino acids His862 and Leu877. Additionally, the substituents on the piperazine nitrogen projected onto a key subpocket consisting of Asp766, Leu769, and Asp770 in PARP-1. Leu769 is replaced by Gly338 in PARP-2, and so this was used as rational for PARP-1 selectivity. Johannes and colleagues (2021[30]) attached various aryl piperazines to an 8-chloroquinazolinone core and found that the interactions between 1) the piperazine moiety and His862 through water molecules and 2) the imidazole moiety and Asp770 via a hydrogen bond resulted in selectivity toward PARP-1 (Johannes et al., 2021[30]). Yu and colleagues (2022[83]) used the key amino acid differences between PARP-1 (Gln759, Glu763, and Asp766) and PARP-2 (Gln324, Ser328, and Gln332) and further modified rucaparib to obtain increased selectivity of PARP-1 inhibitors (Yu et al., 2022[83]). They discovered that Y49 showed excellent selectivity (IC_50_ of PARP-1 and PARP-2, 0.96 nM and 61.90 nM, respectively). Molecular docking demonstrated hydrogen bond formation between the amino group of 4-aminopiperidine-1-yl with Glu763 and Asp766 in PARP-1, whereas 4-aminopiperidine-1-yl caused steric hindrance in PARP-2. Thus, they suggested that nitrogen-containing basic substituents were required to fit into the hydrophilic pocket formed by acidic amino acids around the AD site.

### Model deployment as web server

To facilitate accessibility for non-chemoinformatic scientists who intend to determine whether their compounds have PARP-1 inhibitory activity, a public web server was created. Thus, the predictive model, PARP1pred, is available at https://parp1pred.streamlitapp.com.

**Figure 9:**
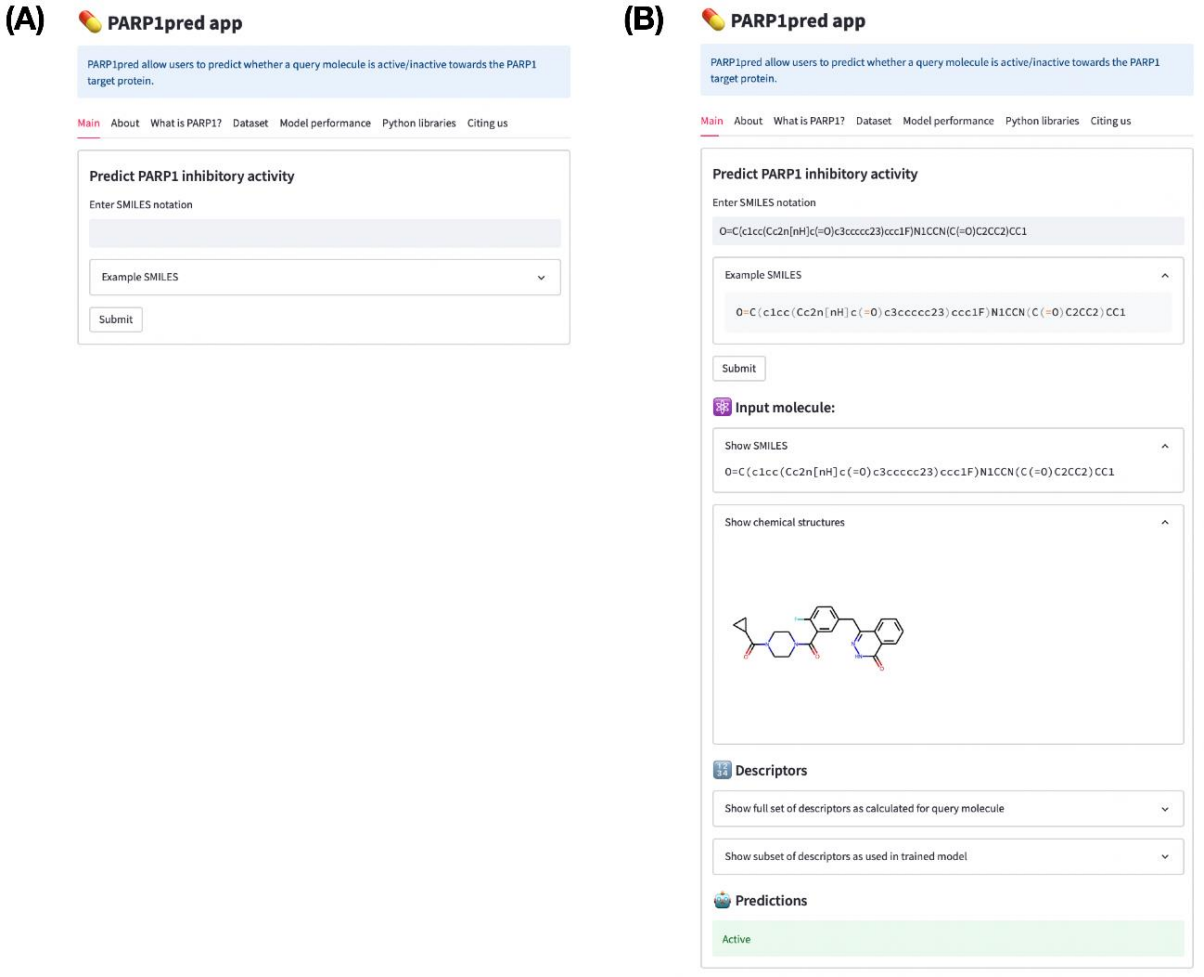
Screenshot of the PARP1pred webserver before (A) and after (B) entering the SMILES input. Notice that after submission of the SMILES notation the corresponding molecular fingerprints are computed whereby the trained predictive model is applied to classify the query molecule as active or inactive. In this case, the query molecule is classified to be active.

Briefly, the PARP1pred web server uses SMILES as the input for the query compound. PadelPy is used to convert SMILES to PubChem fingerprints, which are then used as an input to trained classification models whose outputs are reported as active or inactive (Figure 9(Fig. 9)).

## Conclusion

In the era of precision medicine, targeting of DNA repair is effective in killing cancer cells. PARP-1 plays a role in DNA damage and repair, and is a well-known target for cancers with *BRCA1/2* mutations. Several drugs targeting PARP-1 have been FDA approved; however, accessing such targeted drugs is problematic because of their high cost, particularly in middle- and low-income countries. Thus, advancements in drug development would contribute to the alleviation of such access constraints. In this study, computer-aided drug design was used to understand the relationship between the chemical structures of inhibitors and PARP-1 through the QSAR building model. Understanding such relationships will facilitate rational drug design to effectively target PARP-1. Our study retrieved a set of biological activities from the ChEMBL database that contained 2018 non-redundant compounds. A PubChem fingerprint-based random forest classification model from an oversampling approach was built to predict PARP-1 activity. Gini index calculation revealed the important features in the random forest model, which included aromatic/cyclic/heterocyclic moieties and nitrogen-containing fingerprints, and ether/aldehyde/alcohol moieties. Additionally, a detailed examination of the structure-activity relationship revealed that hydrophobic interactions and hydrogen bonding networks with nitrogen-containing scaffolds are critical for developing PARP-1 inhibitors. As a result, this insight provides a framework for data-driven PARP-1 inhibitor design.

## Notes

Tassanee Lerksuthirat, Aijaz Ahmad Malik (Center of Excellence in Computational Molecular Biology, Faculty of Medicine, Chulalongkorn University, Bangkok 10330, Thailand; E-mail: ajaz_me@hotmail.com) and Chanin Nantasenamat (Streamlit Open Source, Snowflake Inc., USA; E-mail: hellodataprofessor@gmail.com) contributed equally as corresponding author.

## Declaration

### Conflict of interests

All authors declare that there are no conflicts of interest.

### Acknowledgments

We thank Dr. Patipark Kueanjinda for useful discussion on machine learning. We thank Catherine Perfect, MA (Cantab), from Edanz (www.edanz.com/ac), for editing a draft of this manuscript. This project is funded by the National Research Council of Thailand (NRCT) and Mahidol University (NRCT5-TRG63009-04).

## Figures and Tables

**Table 1:**
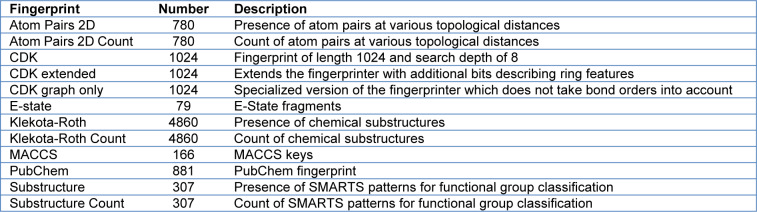
Twelve different sets of fingerprint descriptors derived from the PaDEL-Descriptor software

**Table 2:**
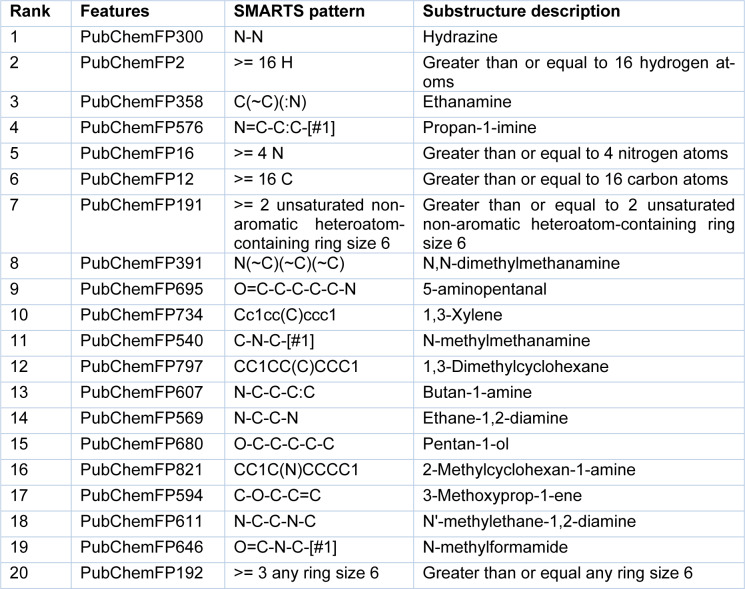
Descriptions of SMARTS patterns and substructures from the top 20 Gini indices
